# Supplementary material for: Hypothermal opto-thermophoretic tweezers
Source: Nat Commun. 2023 Aug 23;14:5133. doi: 10.1038/s41467-023-40865-y (PMC10447564; doi:10.1038/s41467-023-40865-y)
Supplement: Supplementary file 1 — Supplementary Information [file 41467_2023_40865_MOESM1_ESM.pdf]

Supplementary Information

Hypothermal opto-thermophoretic tweezers

Authors: Pavana Siddhartha Kollipara[1], Xiuying Li[2], Jingang Li[3,4], Zhihan Chen[3], Hongru Ding[1], Youngsun Kim[3], Suichu Huang[5], Zhenpeng Qin[2,6,7,8], Yuebing Zheng[1,3,*]

Affiliations:

[1]Walker Department of Mechanical Engineering, The University of Texas at Austin, Austin, Texas, 78712, USA.

[2]Department of Mechanical Engineering, The University of Texas at Dallas, Richardson, Texas, 75080, USA

[3]Materials Science and Engineering Program and Texas Materials Institute, The University of Texas at Austin, Texas, 78712, USA

[4]Laser Thermal Laboratory, Department of Mechanical Engineering, University of California, Berkeley, California 94720, USA

[5]Key Laboratory of Micro-Systems and Micro-Structures Manufacturing of Ministry of Education and School of Mechatronics Engineering, Harbin Institute of Technology, Harbin 15001, China

[6]Department of Bioengineering, The University of Texas at Dallas, Richardson, Texas, 75080, USA

[7]Department of Biomedical Engineering, The University of Texas Southwestern Medical Center, Dallas, Texas, 75390, USA

[8]Center for Advanced Pain Studies, The University of Texas at Dallas, Richardson, Texas, 75080, USA

[*]Correspondence to: zheng@austin.utexas.edu

Supplementary Figures:

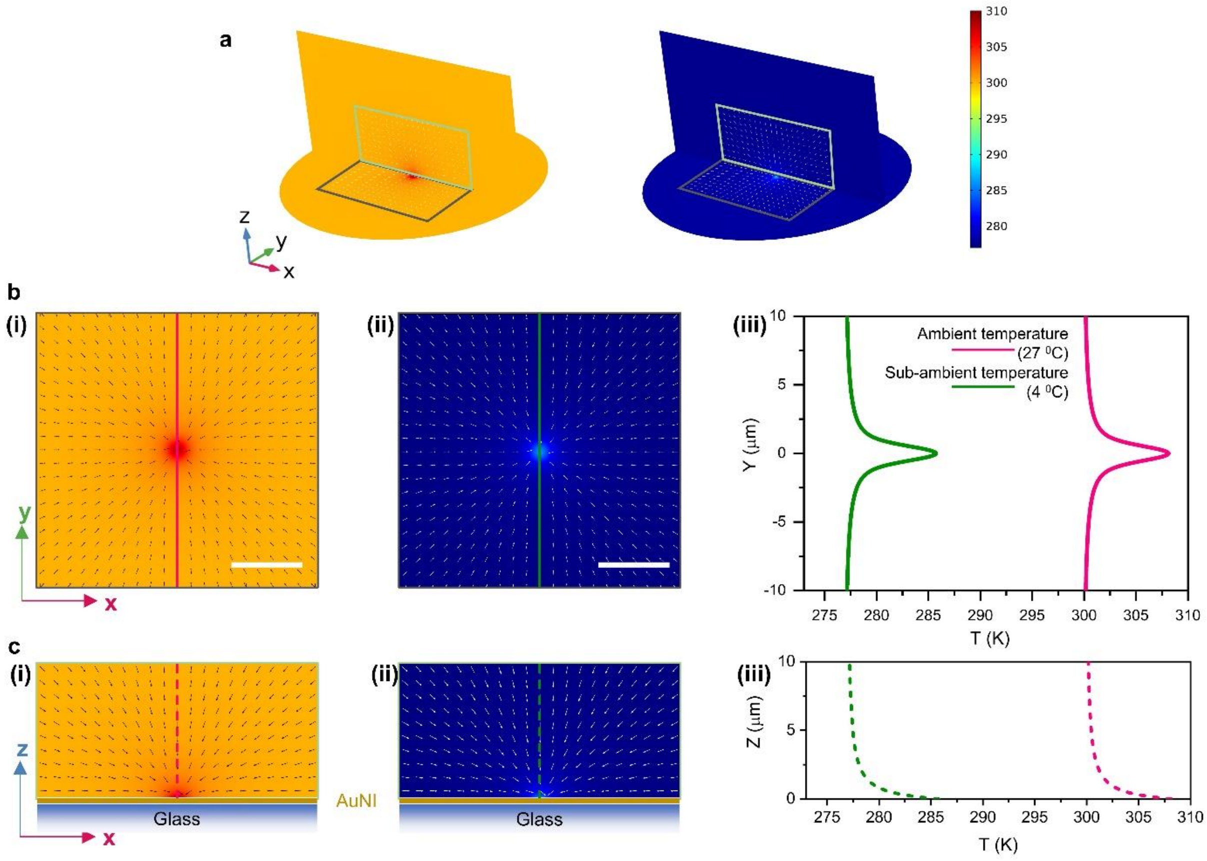

Supplementary Figure 1: Temperature distribution due to laser heating thin AuNI on glass substrate: a) Perspective view of laser heating the sample at ambient temperature (left panel, 27 ^{0}C) and sub-ambient temperature (right panel, 4 ^{0}C). Color bar indicates the temperature in K. The dark gray and green rectangles indicate the substrate and perpendicular to the substrate respectively. b,c) Temperature distribution along the substrate (b) and vertical to the substrate (c) at ambient (i) and sub-ambient temperature (ii). The one-dimensional temperature profiles highlighted using lines on (i) and (ii) and indicated in (iii). Laser power: 0.45 mW. Scale bars: 5 μm.

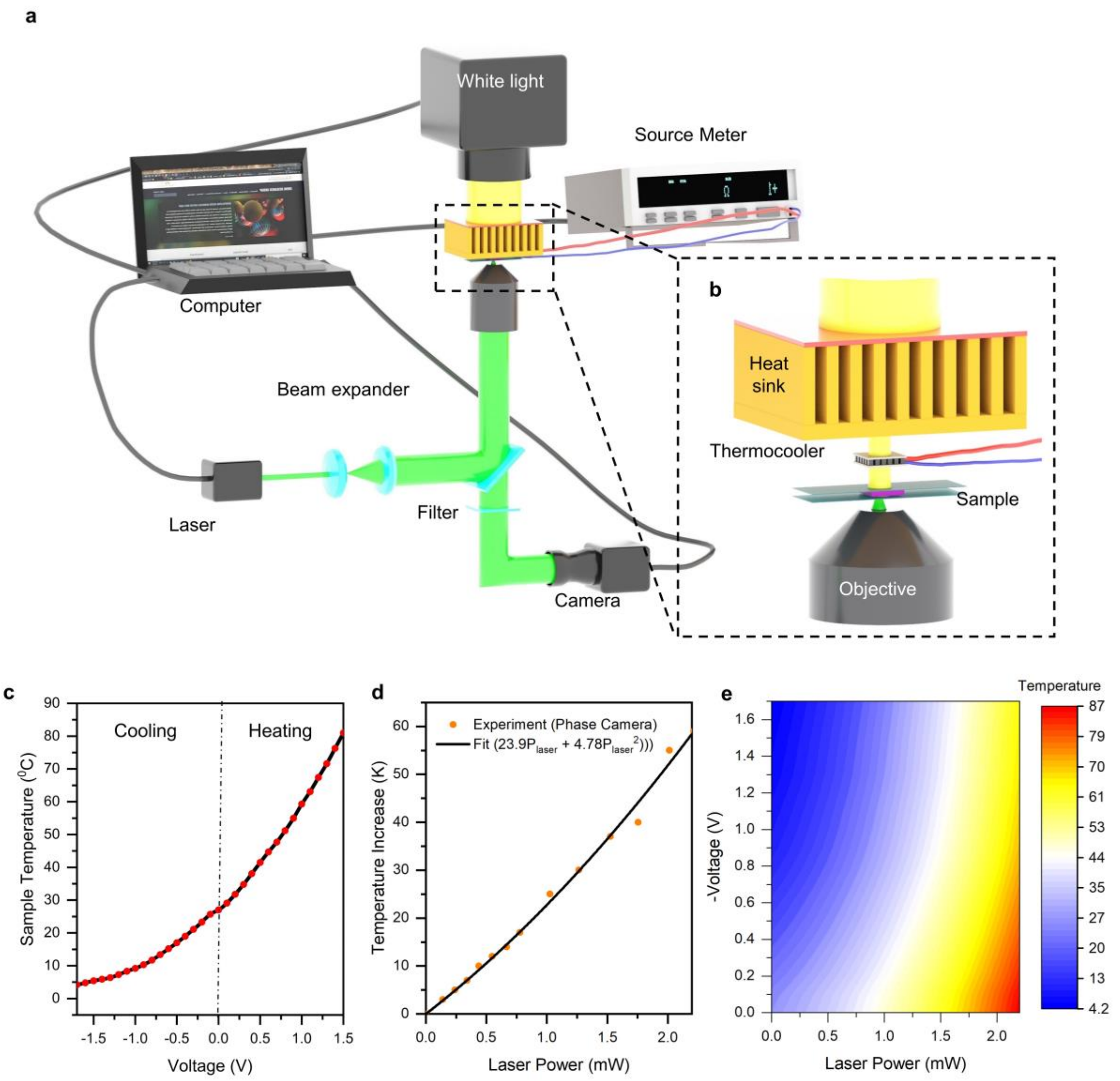

Supplementary Figure 2: Experimental Setup and Temperature control of sample and the trap: a) The laser is expanded using a beam expander and directed towards the sample through an objective (40X magnification). A white lamp is used to illuminate the sample. The transmitted white light and the reflected laser at the sample is directed to a charge-coupled device (CCD) camera. The source meter is used to control the Peltier thermoelectric device (thermocooler). The laser, camera, white light, objective, and sample position is controlled using a computer. b) Zoomed-in view shows the exploded view of the objective, aqueous sample over the objective, thermocooler and its heat sink. c) The sample temperature as a function of applied voltage across the thermocooler demonstrates that the device can be used for both heating and cooling. d) The maximum localized temperature increment as a function of incident laser power measured by a phase camera (introduced instead of CCD camera only for this experiment). e) The maximum temperature of the laser trap as a function of thermocooler voltage and laser power.

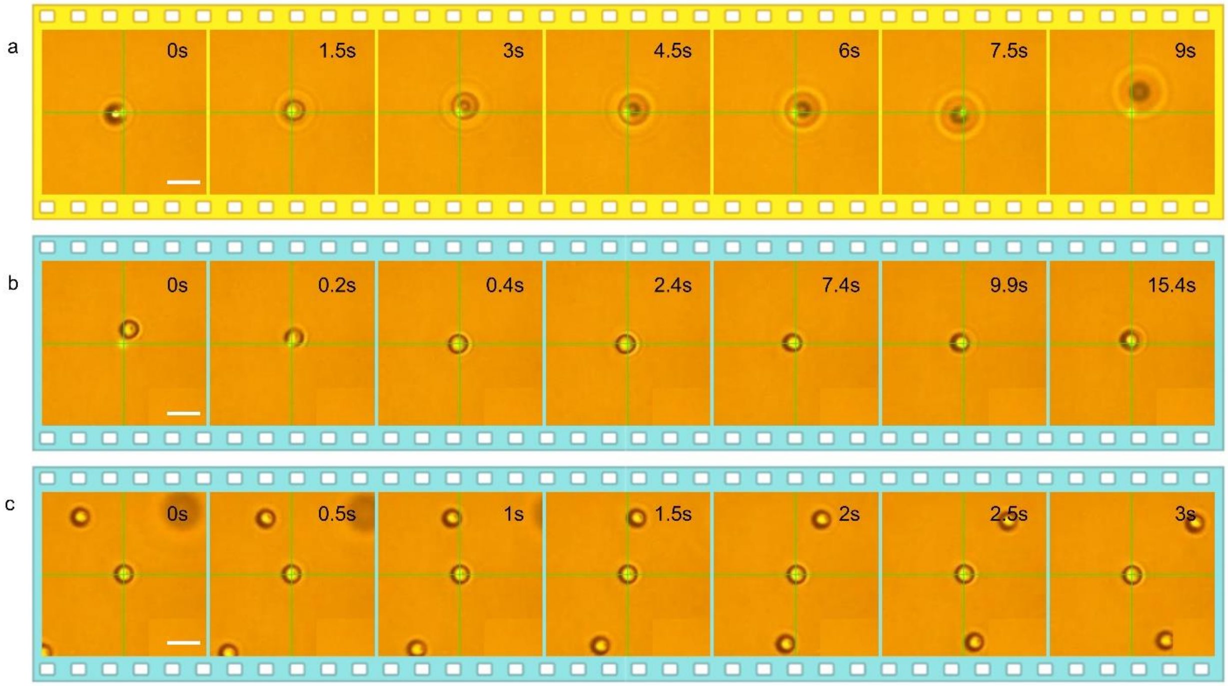

Supplementary Figure 3: Trapping and Manipulation of 2.66 μm COOH-functionalized PS particles: a) Repulsion of a PS-COOH 2.66 μm particle under 532 nm laser excitation at ambient temperature of 27 ^{0}C. Optical repulsion force dominates and moves the particles away from the laser beam. b) Attraction and trapping of the particle at sub-ambient temperature of 10 ^{0}C. c) Manipulation of the particle across two particles at sub-ambient temperature of 10 ^{0}C. Laser power is 0.14 mW, Scale bar: 5 μm.

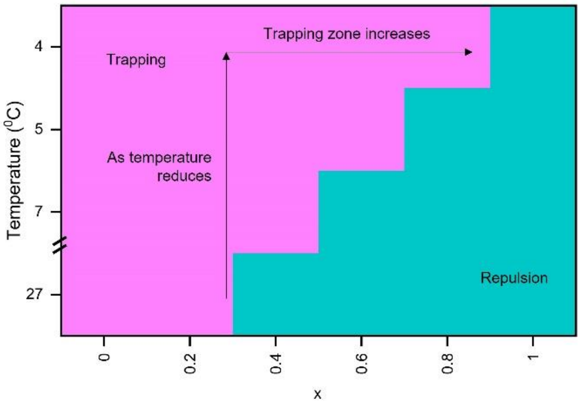

Supplementary Figure 4: Trapping vs Repulsion of 1 μm PS particles in varying composition of 3 mM $NaCl_xOH_{1-x}$: As the temperature reduces, the trapping zone increases considerably. At ambient temperature (27 ^{0}C), trapping is observed only in ~ 20% of the compositions (x $\leq$ 0.2), however, reducing the ambient temperature to 4 ^{0}C increasing the trapping range to ~ 80% of the compositions (x $\leq$ 0.8).

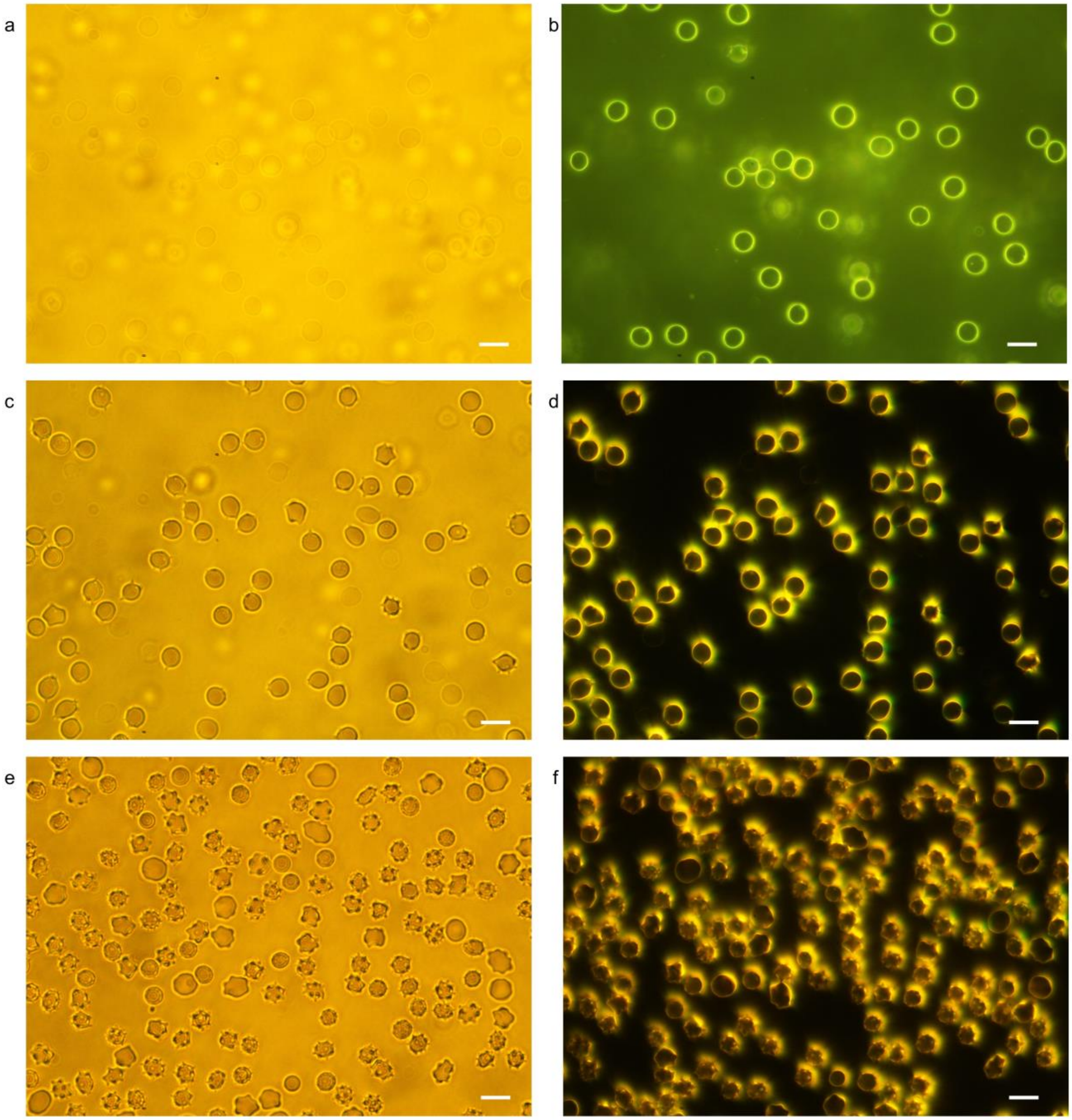

Supplementary Figure 5: **Erythrocytes in different tonicities**: Bright field (a,c,e) and dark field (b,d,f) optical microscopy images at erythrocytes in hypotonic, isotonic, hypertonic PBS solutions. Scale bar: 10 µm.

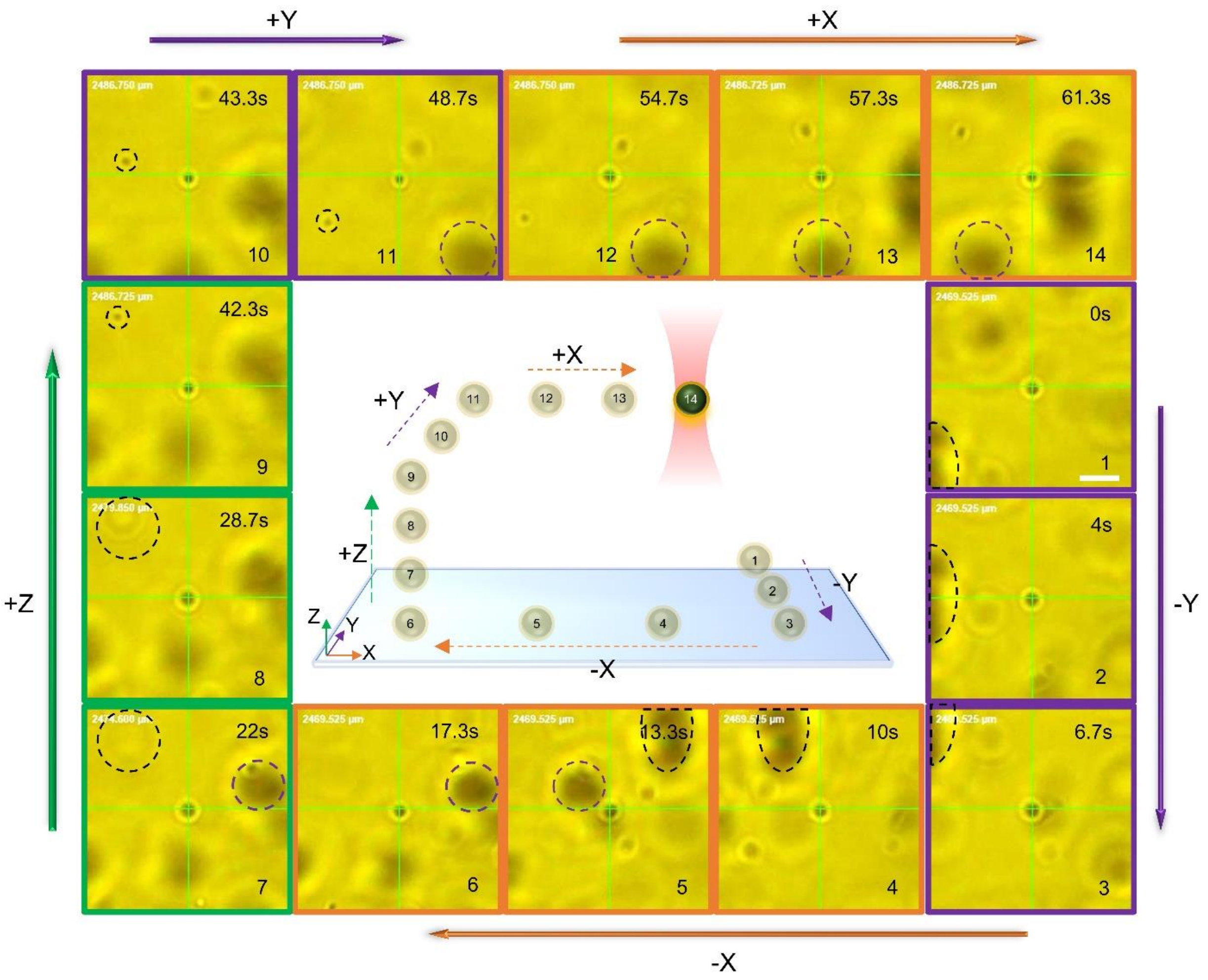

Supplementary Figure 6: Three-dimensional manipulation of plasmonic vesicles: Each panel shows the trapped vesicle at the center of crosshairs. The sequence of images is shown on the right bottom corner of each panel and the dashed curves represent the reference particles/clusters. The panel is first moved along the Y direction (panels: 1-3, purple border), followed by X direction manipulation (panels: 4-6, orange border). The vesicle is then moved vertically upwards to a different plane until the defocused reference vesicle is moved onto the focus plane (panels: 7- 9). The vesicle is later moved in Y direction (panels: 10,11) and X direction (panels: 12-14) at the same Z position. Laser power: 0.67mW. Scale bar: 5 μm.

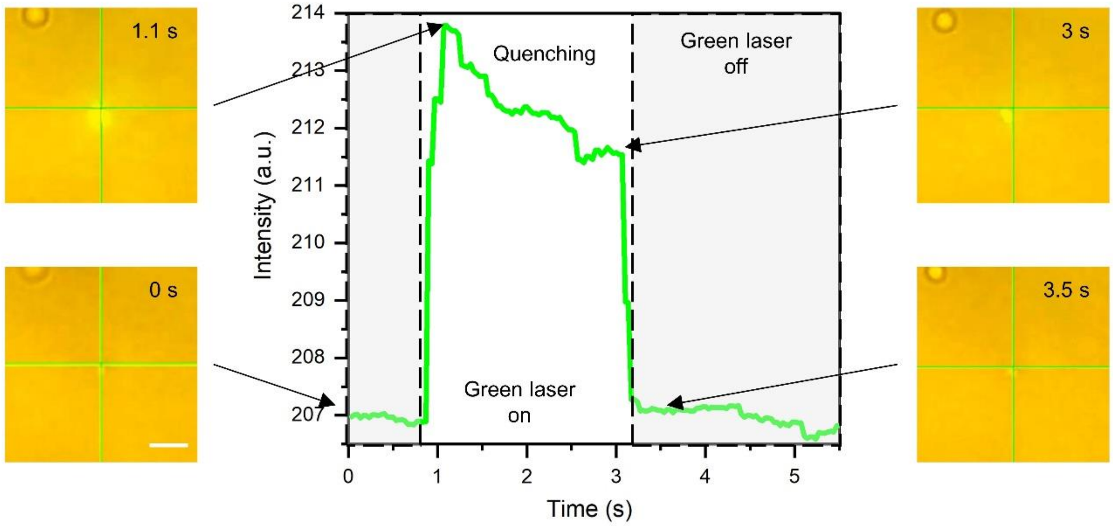

Supplementary Figure 7: Fluorescent intensity of plasmonic vesicles during rupture: The vesicle is manipulated using the red laser and positioned. The green laser is then focused on the trapped vesicle at 1.1 s to rupture and release the cargo. Because of fluorescent quenching, the intensity gradually decreases. The green laser is then switched off indicated by the sudden drop in the intensity measurement. Scale bar: 5 μm.

Supplementary Notes:

Supplementary Note 1: Thermophoretic force on microparticles

The thermophoretic drift velocity ($\boldsymbol{u}_{\mathbf{th}}$) of the particle is directly proportional to the mean temperature gradient ($\boldsymbol{\nabla T}$) experienced by the microparticle, and the proportional constant known as the thermo-diffusion coefficient (D_T).

$$\boldsymbol{u}_{\mathbf{th}} = -D_T \boldsymbol{\nabla T} \tag{S1}$$

D_T intricately depends on several parameters such as temperature, colloidal concentration, electrolyte and surfactant type and concentration, polymers, and so on. D_T is related to the Ludwig-Soret coefficient (S_T) as

$$D_T = D.S_T \tag{S2}$$

where D represents the Brownian diffusion coefficient, given in terms of

$$D = C(T_{\mathrm{sub}}).\frac{k_B T_{\mathrm{avg}}}{6\pi\eta(T_{\mathrm{avg}})r_{\mathrm{p}}} \tag{S3}$$

k_B is the Boltzmann constant, T_{avg} is the average temperature of the particle, $\eta(T)$ is the viscosity of the solvent as a function of temperature and r_P is the radius of the particle. The coefficient C represents a correction factor that incorporates distinct phenomena such as the gap between the particle and the substrate, friction due to electrostatic attraction between the particle and substrate, and so on. Since the correction factor arises due to the interactions between the particle and substrate, it strongly depends on the local temperature (T_{sub}) between the particle and the substrate. The Stokes Drag force is given as

$$\boldsymbol{F}_{\mathbf{drag}} = \frac{k_B T_{\mathrm{avg}}}{D}\boldsymbol{u} \tag{S4}$$

Here, $\boldsymbol{u}$ is the escape velocity of the particle, i.e., the limiting velocity of the laser beam where the particle escapes the laser trap. Experimental evaluation of the thermo-diffusion coefficient and the Soret coefficient is mentioned in Supplementary Note 4.

Supplementary Note 2: Temperature controller fabrication

A Peltier thermoelectric device utilizes a voltage across two plates (that encompass a thermoelectric material) to generate a temperature difference across the plates.

$$T_{\text{hot}} - T_{\text{cold}} = c_1 V \quad \text{(S5)}$$

where $T_{\text{hot,cold}}$ are the temperatures of the hot and cold sides of the plate, V is the applied voltage across the two plates, and c_1 is the proportionality constant of the cooler. A commercial Peltier cooler (Laird systems) and a source meter are used to control the temperature difference. However, as the voltage increases, the resistive heating of the thermocooler results in the overall heating of the thermocooler. This leads to the colder side's temperature being approximately equal to the ambient temperature, and the hot side's temperature increasing with varying voltage.

$$T_{\text{cold}} \sim T_{\text{amb}} \ (no\ heat\ sink) \quad \text{(S6)}$$

To overcome this, a commercially available aluminum heat sink (www.amazon.com, Model number: a14111400ux0256) is cut according to the requirements and attached to the hot side of the Peltier device using a thermal interface material/thermal adhesive (www.amazon.com, SOBEIT, SH-thermal tape). The heat sink effectively dissipates the generated heat while maintaining the hot plate temperature closer to the ambient temperature. This is because of the increased surface area due to the heat sink.

$$\begin{gathered} T_{\text{hot}} \sim T_{\text{amb}} \ (with\ heat\ sink) \\ T_{\text{cold}} = T_{\text{amb}} - c_2 V \end{gathered} \quad \text{(S7)}$$

Note that c_1 and c_2 are different proportionality constants corresponding to the Peltier device without and with the attached heat sink respectively. The resultant device is then directly placed on the coverslip which is supported over the objective by a sample holder. The holder holds the coverslip by all its edges to limit bending during temperature changes. The effective temperature of the sample in this

configuration is then measured using an infrared camera as a function of the applied voltage as shown in Supplementary Fig. 2c.

Supplementary Note 3: Evaluation of trapping stiffness on the particle

The trapped particle is tracked using Nikon CCD using a time step of 10 ms (effective timestep while saving videos is 30 ms). The trajectory of the trapped particle is fitted to a gaussian distribution curve, and the standard deviation of the fit σ is used to determine the trapping stiffness as

$$\kappa = \frac{k_B T}{\sigma^2}$$

where κ is the trapping stiffness and σ is the standard deviation of the Gaussian fit for the histogram of X coordinates. Distinct particles were trapped for evaluating the mean and standard deviation of the trapping stiffness.

Supplementary Note 4: Experimental evaluation of Correction factor, Brownian diffusion coefficient, thermo-diffusion coefficient, and Soret Coefficient as a function of temperature

Brownian diffusion coefficient and Correction factor determination

To simultaneously evaluate the thermo-diffusion coefficient (or the Soret coefficient) and the thermophoretic force on the particle, the correction factor $C(T)$ in the equation S3 needs to be evaluated experimentally. SiO_2 particles are dispersed and sonicated in DI water and introduced into the coverslip (Supplementary Fig. 8a). Note that SiO_2 particles are used because they can gradually get settled on the substrate due to their weight giving a consistent opportunity to evaluate the effects of the substrate. Also, SiO_2 particles can be trapped at ambient temperature, which provides a greater working range in terms of temperature study. The sample temperature is then controlled using the temperature controller. The Brownian motion of the particles is recorded using a Nikon CCD camera at a frame rate (δt) of 10 ms. Using image tracking, the trajectory of several particles is obtained as a function of time (Supplementary Fig. 8b). Please note that no laser beam is used to trap these particles. The trajectory is then used to evaluate the diffusion coefficient based on the mean displacement $< \sigma^2 >$ of the particle

$$< \sigma^2 > = 4D(T)\delta t \tag{S8}$$

The obtained Brownian diffusion coefficient $D(T)$ is plotted as a function of temperature in Supplementary Fig. 8c. The correction factor $C(T)$ is then evaluated using equation (S3) using $T_{\text{sub}} = T_{\text{avg}} = T_{\text{sample}}$, to obtain as a function of temperature.

<u>Local temperature and mean temperature of the particle as a function of laser particle distance</u>

Parallelly, temperature simulations are performed to analyze the temperature distribution of the particle situated near the laser beam. This enables us to evaluate the average viscosity of water as a function of mean temperature, and the correction factor as a function of substrate temperature simultaneously.

<u>Thermo-diffusion coefficient and Soret Coefficient as a function of temperature:</u>

For a given laser power and Peltier device voltage, the temperature distribution can be evaluated using a series of experiments and theoretical simulations as highlighted in the previous sections. Using the exact local temperature, and the average temperature distribution of the particle, the net (drag) force in the lateral direction (perpendicular to the laser axis) on the particle is evaluated as

$$F_{\text{th}} = F_{\text{net}} - F_{\text{opt}} = F_{\text{drag}} - F_{\text{opt}} \tag{S9}$$

F_{opt} in the lateral direction is evaluated via simulations in Supplementary Note 5. F_{drag} is experimentally evaluated using Equations (S3) and (S4). The laser-particle distance is set as 0.9 μm from particle manipulation experiments. Therefore, the net thermophoretic force acting on the particle can be evaluated using S9. We can associate a thermophoretic drift velocity to the net thermophoretic force like Stokes drag force as

$$\boldsymbol{F}_{\textbf{th}} = \frac{k_B T}{D} \boldsymbol{u}_{\textbf{th}} = -\frac{k_b T}{D} D_T \boldsymbol{\nabla T} = -k_B T S_T \boldsymbol{\nabla T} \tag{S10}$$

Please note that the $\boldsymbol{u_{th}}$ is different from $\boldsymbol{u}$, the former being the thermophoretic drift velocity and the latter being the escape velocity ($u_{th} = u$ when optical force = 0). Here, we use a force-based approach to

include the non-zero contribution of the optical force, which may be significant, while extending this work to other metallic and light-absorbing particles. The Soret coefficient can then be evaluated as a function of temperature and is displayed in Supplementary Fig. 8d. The variation in Soret coefficient arises from the variation in Brownian diffusion coefficient and can be limited by using an ultrafast camera and a good graphics card to record videos at a faster frame rate. The resultant Soret Coefficient follows the standard empirical relation:

$$S_T = S_{T,\infty}\left(1 - e^{\frac{T^* - T}{T_0}}\right) \quad \text{(S11)}$$

$S_{T,\infty}$, T^* and T_0 are fitting parameters and T^* is the transition temperature between the thermophobic and thermophilic nature of the particle. The transition temperature for SiO_2 particles is estimated around $32.5 \pm 2.1\ ^0$C.

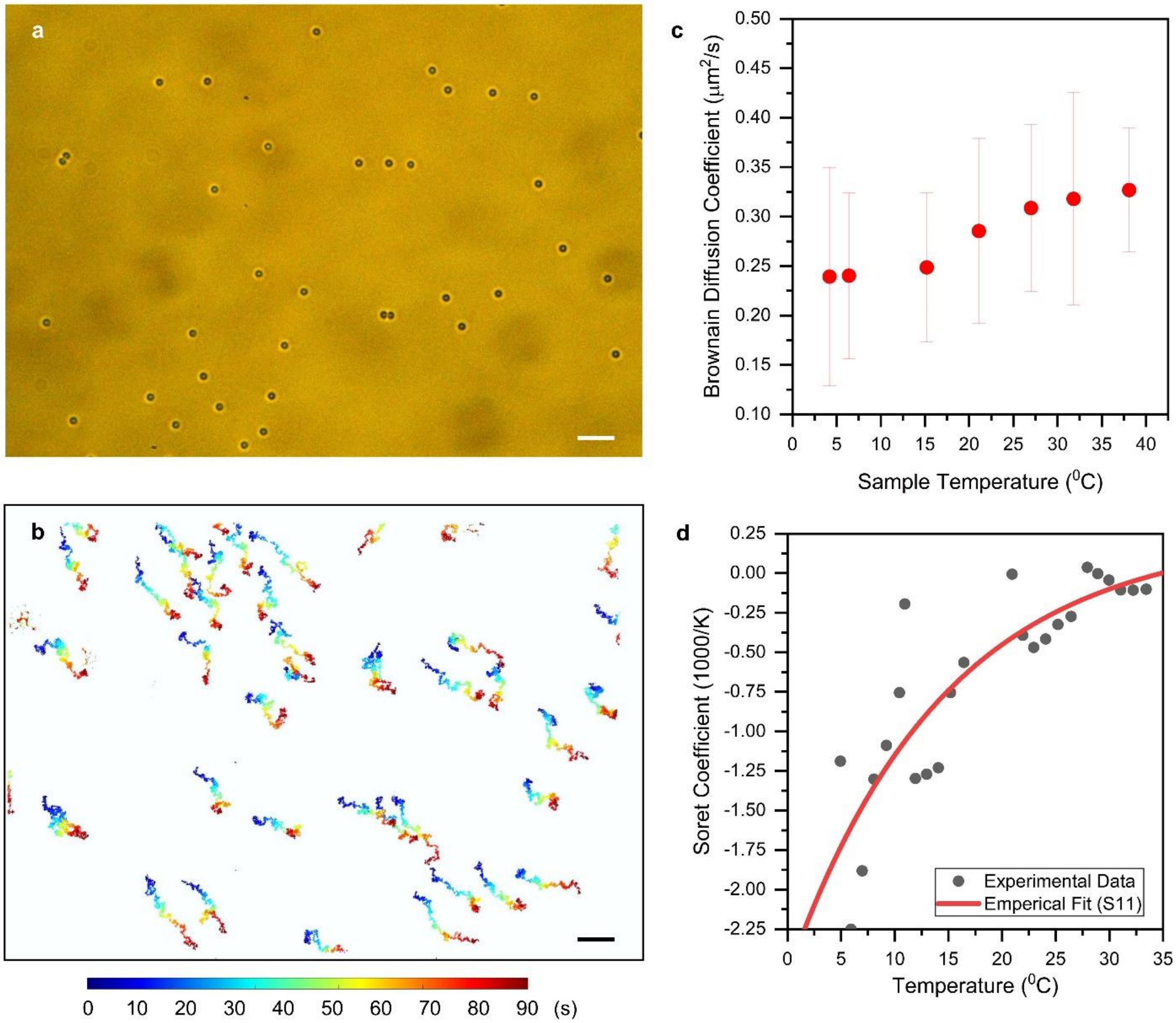

Supplementary Figure 8: Experimental derivation of Soret Coefficient as a function of temperature: a) Brightfield optical microscopic image of 1.96 SiO_2 particles dispersed in DI water. b) Trajectories of particles as a function of time. c) Experimental valuation of Brownian Diffusion coefficient at varying temperatures. Data are presented as mean values $\pm$ standard deviation obtained from analysis of the 29 different trajectories in Figure 2b. d) Soret Coefficient of SiO_2 particles in DI water as a function of temperature. Scale bars: 10 μm (a,b).

Supplementary Note 5: Optical force on microparticles

Optical forces on SiO_2 1.96 µm particles are evaluated using Ansys Lumerical FDTD (ver. 2021 R2.5). The refractive index of SiO_2 particles is assumed as 1.46, water as 1.33, and glass coverslip as 1.46. Optical force is evaluated at 532 nm, with a beam radius of 840 nm (measured from experiments) at the interface of glass coverslip and water. The total domain size is set to 3000 µm in either direction to encompass the complete laser beam. A mesh grid of 3 nm is used for meshing the particle. The setup is run on clusters provided by the Texas Advanced Computing Center, using 5 x 32 cores. Since micrometer-sized particles were used in this study, we employed the Maxwell Stress Tensor (MST) methodology to evaluate the electric field and magnetic field around the particle using E-field monitors, using Lumerical's script for evaluation of optical forces. The space between the monitor and the particle in all directions is set to three mesh cells (~9 nm). Supplementary Fig. 9 shows the optical forces perpendicular to and along the laser beam propagation axis respectively. Negative force indicates attraction and positive force indicates repulsion.

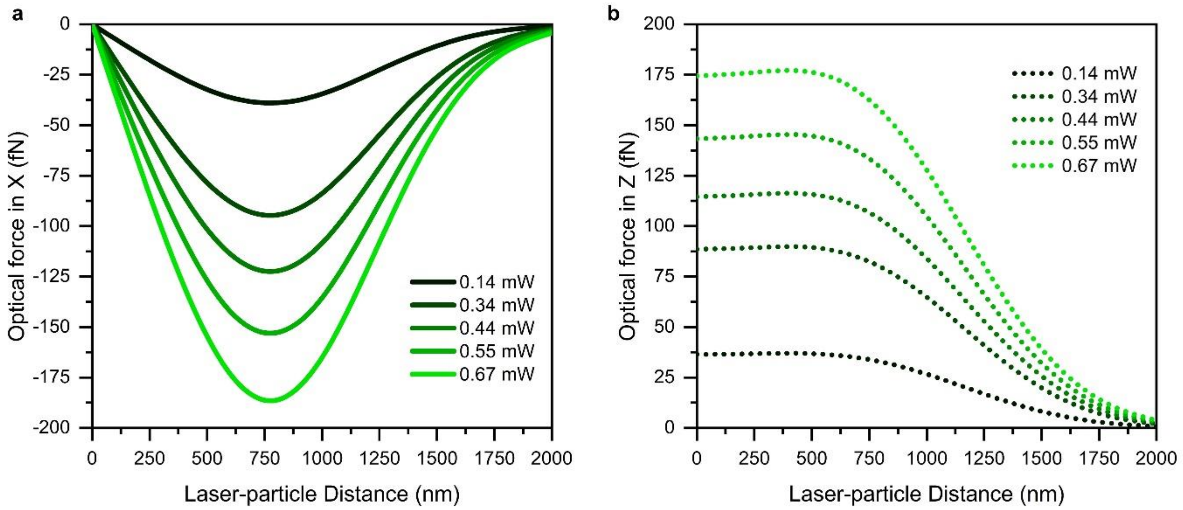

Supplementary Figure 9: Optical force on SiO_2 microparticles by 532 nm laser beam at varying powers (a) perpendicular to the laser beam axis (X direction) and (b) along the laser beam axis (Z direction)

Supplementary Tables:

Supplementary Table S1: Literature survey of aqueous solutions of diverse particles exhibiting temperature-dependent Soret coefficient and following the empirical law $\left(S_T = S_{T,\infty}\left(1 - e^{\frac{T^*-T}{T_0}}\right)\right)$

Sl.No	Particle	Size/conc.	Solvent	Temperature	Soret coefficient (K^{-1})	Reference	Follows empirical law?
1	Pullulan	5g/mL	Water	20	-0.06	[1]	Yes
				50	0.02		
2	PS	90 nm	5 mM NaCl in water	27	2	[2]	Yes
				47	4.6		
			5 mM NaOH in water	41-57	-1.25		No
3	DNA	5-50 bases	Water	5	-0.075 – -0.025	[3]	Yes
				70	0-0.05		
4	$CoFe_2O_3$ core, γ-Fe_2O_3 shell	13.6 nm	10 mM HNO_3	20	-3.25	[4]	Yes
				55	-0.5		
5	KI	1 mol/kg	Water	15	-0.001	[5]	Yes
				45	+0.001		
	NaI			15	-0.001		
				45	+0.0015		
	LiI			15	-0.004		
				45	-0.002		
6	**PS**	2.5 μm	Water	10	-80	[6]	Yes
				37	250		
	Melamine	1.35 μm	Water	10	-90		
				57	15		
7	PS	30 nm	4 mM NaCl	5	-0.02	[6]	Yes
				40	0.24		
8	Streptavidin	39 g/L	Water	10	-0.028	[7]	Yes
				50	0.02		
	Biotin	0.94 g/L	Water	10	0.005		
				50	0.01		
9	Gold NPs	28 nm	Water	28	-0.01	[8]	Yes
				40	0.02		
10	PS-COOH	26 nm	Water (pH 6.5)	10	-0.35	[9]	Yes
				60	0.3		
11	WT lysozyme	-	Water	10	-0.01	[9]	Yes
				50	0.01		
12	PS-COOH	123 nm	1mM Tris-HCl buffer	10	-0.6	[10]	Yes
				45	1.5		
13	Lysozyme	7 g/l	400 mM NaCl	5	-0.02	[11]	Yes
				35	0.008		
14	Vesicles – DOPC, DPPC (and others)	1 μm		5	-0.05	[12]	Yes
				55	0.45		

Supplementary References:

1 Kishikawa, Y., Wiegand, S. & Kita, R. Temperature Dependence of Soret Coefficient in Aqueous and Nonaqueous Solutions of Pullulan. *Biomacromolecules* **11**, 740-747, doi:10.1021/bm9013149 (2010).

2 Eslahian, K. A., Majee, A., Maskos, M. & Würger, A. Specific salt effects on thermophoresis of charged colloids. *Soft Matter* **10**, 1931, doi:10.1039/c3sm52779d (2014).

3 Reichl, M., Herzog, M., Götz, A. & Braun, D. Why Charged Molecules Move Across a Temperature Gradient: The Role of Electric Fields. *Physical Review Letters* **112**, doi:10.1103/physrevlett.112.198101 (2014).

4 Sehnem, A. L. *et al.* Temperature dependence of the Soret coefficient of ionic colloids. *Physical Review E* **92**, doi:10.1103/physreve.92.042311 (2015).

5 Mohanakumar, S., Kriegs, H., Briels, W. J. & Wiegand, S. Overlapping hydration shells in salt solutions causing non-monotonic Soret coefficients with varying concentration. *Physical Chemistry Chemical Physics* **24**, 27380-27387, doi:10.1039/d2cp04089a (2022).

6 Helden, L., Eichhorn, R. & Bechinger, C. Direct measurement of thermophoretic forces. *Soft Matter* **11**, 2379-2386, doi:10.1039/c4sm02833c (2015).

7 Niether, D. *et al.* Thermophoresis: The Case of Streptavidin and Biotin. *Polymers* **12**, 376, doi:10.3390/polym12020376 (2020).

8 Shakib, S. *et al.* Microscale Thermophoresis in Liquids Induced by Plasmonic Heating and Characterized by Phase and Fluorescence Microscopies. *The Journal of Physical Chemistry C* **125**, 21533-21542, doi:10.1021/acs.jpcc.1c06299 (2021).

9 Putnam, S. A., Cahill, D. G. & Wong, G. C. L. Temperature Dependence of Thermodiffusion in Aqueous Suspensions of Charged Nanoparticles. *Langmuir* **23**, 9221-9228, doi:10.1021/la700489e (2007).

10 Braibanti, M., Vigolo, D. & Piazza, R. Does Thermophoretic Mobility Depend on Particle Size? *Physical Review Letters* **100**, doi:10.1103/physrevlett.100.108303 (2008).

11 Piazza, R., Iacopini, S. & Triulzi, B. Thermophoresis as a probe of particle–solvent interactions: The case of protein solutions. *Phys. Chem. Chem. Phys.* **6**, 1616-1622, doi:10.1039/b312856c (2004).

12 Talbot, E. L., Kotar, J., Parolini, L., Di Michele, L. & Cicuta, P. Thermophoretic migration of vesicles depends on mean temperature and head group chemistry. *Nature Communications* **8**, 15351, doi:10.1038/ncomms15351 (2017).
