# Supplementary material for: Hypothermal opto-thermophoretic tweezers
Source: Nat Commun. 2023 Aug 23;14:5133. doi: 10.1038/s41467-023-40865-y (PMC10447564; doi:10.1038/s41467-023-40865-y)
Supplement: Supplementary file 3 — Description of Additional Supplementary Files [file 41467_2023_40865_MOESM3_ESM.pdf]

Description of Additional Supplementary Files:

Supplementary Movie 1: Repulsion and trapping of 1 μm PS particles in DI water at ambient and sub-ambient temperatures respectively

Supplementary Movie 2: Repulsion and trapping of 2.66 μm PS-COOH particles in DI water at ambient and sub-ambient temperatures respectively

Supplementary Movie 3: Repulsion and trapping of 1.96 μm SiO_2 particles in DI water at 31.6 ^{0}C and sub-ambient temperatures respectively

Supplementary Movie 4: Repulsion and trapping of 1 μm PS particles in 1mM $NaCl_{0.2}NaOH_{0.8}$ solution at ambient and sub-ambient temperatures respectively

Supplementary Movie 5: Rupture and trapping of isotonic erythrocytes in PBS 0.5X at ambient and sub-ambient temperatures respectively

Supplementary Movie 6: Repulsion and trapping of hypotonic erythrocytes in PBS 0.3X at ambient and sub-ambient temperatures respectively

Supplementary Movie 7: Repulsion and trapping of hypertonic erythrocytes in PBS 0.7X at ambient and sub-ambient temperatures respectively

Supplementary Movie 8: Repulsion and trapping of plasmonic vesicles at ambient and sub-ambient temperatures respectively

Supplementary Movie 9: Three-dimensional manipulation of plasmonic vesicles at sub-ambient temperature

Supplementary Movie 10: Manipulation followed by controlled drug release of plasmonic vesicles at sub-ambient temperature
